# Mycotoxin Assessment in Minimally Processed Traditional Ecuadorian Foods

**DOI:** 10.3390/foods14152621

**Published:** 2025-07-26

**Authors:** Johana Ortiz-Ulloa, Jorge Saquicela, Michelle Castro, Alexander Cueva-Chamba, Juan Manuel Cevallos-Cevallos, Jessica León

**Affiliations:** 1 Department of Biosciences, Faculty of Chemical Sciences, University of Cuenca, Av. 12 de Abril s/n Cdla. Universitaria, Cuenca P.O. Box 01.01.168, Ecuador; jorge.saquicela@ucuenca.edu.ec (J.S.); evelyn.castro@ucuenca.edu.ec (M.C.); alexander.cueva@ucuenca.edu.ec (A.C.-C.); jessica.leonv@ucuenca.edu.ec (J.L.); 2 Centro de Investigaciones Biotecnológicas del Ecuador (CIBE), Escuela Superior Politécnica del Litoral, Campus Gustavo Galindo Km. 30.5 Vía Perimetral, Guayaquil P.O. Box 09.01.863, Ecuador; jmceva@espol.edu.ec

**Keywords:** mycotoxins, Ecuador, traditional crops, ancestral crops, whole grains

## Abstract

Nowadays, there is special interest in promoting the consumption of ancestral crops and minimally processed foods with high nutritional value. However, besides nutritional issues, safety assessments must be addressed. This study aimed to evaluate mycotoxin contamination in five minimally processed traditional Ecuadorian foods: ochratoxin A (OTA), fumonisin B_1_ (FB_1_), and aflatoxins (AFs) in brown rice, lupin, and quinoa; OTA, FB_1_, and deoxynivalenol (DON) in whole-wheat flour; and OTA and AFs in peanuts. Samples (45 samples of peanuts and whole-wheat flour, 47 of brown rice, 46 of quinoa, and 36 of lupin) were collected from local markets and supermarkets in the three most populated cities in Ecuador. Mycotoxins were determined by RP-HPLC with fluorescence and detection. Results were compared with the maximum permitted levels (MPLs) of European Regulation 2023/915/EC. Overall contamination reached up to 59.8% of the analyzed samples (38.4% with one mycotoxin and 21.5% with co-occurrence). OTA was the most prevalent mycotoxin (in 82.6% of quinoa, 76.7% of whole-wheat flour, 53.3% of peanuts, 48.6% of lupin, and 25.5% of brown rice), and a modest number of quinoa (17%) and lupin (5.7%) samples surpassed the MPLs. DON was found in 82.2% of whole-wheat flour (28.9% > MPL). FB_1_ was detected in above 25% of brown rice and whole-wheat flour and in 9% of the quinoa samples. FB_1_ levels were above the MPLs only for whole-wheat flour (17.8%). AFB_1_ and AFG_1_ showed similar prevalence (about 6.5 and 8.5%, respectively) in quinoa and rice and about 27% in peanuts. Overall, these findings underscore the importance of enhancing fungal control in the pre- and post-harvest stages of these foods, which are recognized for their high nutritional value and ancestral worth; consequently, the results present key issues related to healthy diet promotion and food sovereignty. This study provides compelling insights into mycotoxin occurrence in minimally processed Ecuadorian foods and highlights the need for further exposure assessments by combining population consumption data.

## 1. Introduction

Mycotoxins are fungal secondary metabolites that affect the quality and safety of a wide array of food commodities [1,2,3]. Mycotoxin contamination can arise at various stages of the food value chain, particularly during pre- and post-harvest. In the pre-harvest phase, factors such as suitable weather conditions, susceptibility to fungal colonization, poor crop rotation, suboptimal planting density, nutrient deficiencies, and drought stress significantly increase the risk of contamination. For example, *Fusarium* spp. infect cereals during growth under humid conditions and can produce fumonisins (FB_1_) and deoxynivalenol (DON), while *Aspergillus* spp., which thrive in warm and humid climates, colonize legumes and grains, leading to aflatoxin (AF) accumulation. Additionally, ochratoxin A (OTA) can originate from field infection or improper harvesting practices. Post-harvest, inadequate drying, high storage humidity, pest infestation, and mechanical damage to grains (e.g., shelling or milling) can further promote fungal proliferation and mycotoxin accumulation [4,5].

Currently, more than 400 types of mycotoxins have been identified. However, only 10 to 12 are considered of the highest public health concern due to their acute and chronic toxic effects after ingestion [6,7,8]. Among the most concerning mycotoxins, aflatoxin B_1_ (AFB_1_) is a potent human carcinogen associated with liver cancer, hepatitis, and immunosuppression; OTA has nephrotoxic, hepatotoxic, and carcinogenic effects and has been linked to endemic nephropathy; FB_1_ is associated with esophageal cancer, liver damage, and neural tube defects, and DON is linked to intestinal inflammation, vomiting, immune suppression, hepatotoxicity, nephrotoxicity, and reproductive toxicity [2,9]. Moreover, there is a high probability of simultaneous dietary exposure to multiple mycotoxins and therefore to synergistic and cumulative health effects [10,11]. Although in vivo data on the toxic effects of mycotoxin co-occurrence are very limited, it has been suggested that mycotoxins with similar mode of action would be expected to have additive effects [12,13]. Some in vitro studies have shown that mycotoxin combinations can produce cytotoxicity by enhancement or synergism [14,15,16], neurodegeneration [17], and other pathogenic pathways [18,19].

It is estimated that more than 25% of the world crop production is contaminated with mycotoxins [20], with annual food losses of about 1 billion metric tons equivalent to 5–10% of the world’s total food [21,22]. The most common commodities that are susceptible to mycotoxin contamination are cereals, cereal-based products, legumes, and pseudocereals [3,23,24]. These commodities are also considered highly nutritious foods, especially if they are consumed as whole grains [25,26]. For instance, lupin is a legume rich in protein, fiber, and essential vitamins [27]. Quinoa, considered a superfood, provides high levels of protein, fiber, lipids, essential amino acids, and micronutrients [28]. Whole-grain rice (also known as brown rice) and whole-wheat flour are also nutritious staples that deserve more attention in the Andean diet. These foods retain their bran layer, providing additional fiber and nutrients compared with their polished/refined counterparts [29]. Peanuts are legumes used as staple grain too, being a valuable source of energy and lipids, as well as antioxidants, minerals, and vitamins [30].

In Ecuador, these foods have been used as staples of local and indigenous diets for decades [31,32]. Despite their numerous nutritional benefits, these foods have relatively low consumption rates due to changes in dietary preferences, nutritional transitions, and increased accessibility to processed foods [33]. On the other hand, Ecuador has remarkable climatic diversity due to its geographical location in the equatorial zone and its complex topography. Coastal and Amazon regions are characterized by warm and humid climates, with average temperatures between 25 and 28 °C, while the Andean region exhibits a temperate climate, with temperatures ranging between 10 and 18 °C. These environmental conditions favor the growth of toxigenic fungi, which in turn facilitates mycotoxins’ synthesis in various crops and food products, in the pre- and post-harvest stages, especially in grains and cereals [34,35].

There is limited scientific information on the presence of mycotoxins in minimally processed traditional Ecuadorian foods. Although the regional consumption of these foods is rather insufficient, their consumption should be encouraged due to their importance in nutrition and food sovereignty [36]. However, strategies towards promoting these nutritious foods should also consider the prior identification and addressing of potential risks of food contamination [37]. In this sense, there is the need to generate reliable analytical data on mycotoxin contamination in traditional Ecuadorian foods that could contribute to the design of mitigation strategies and their safe promotion.

In this study, the most recognized mycotoxins of public health concern, i.e., AFs (AFB_1_, AFB_2_, AFG_1_, and AFG_2_), OTA, FB_1_, and DON, were assessed in five traditional minimally processed Ecuadorian foods (whole-wheat flour, brown rice, lupin, quinoa, and peanuts) of interest for the promotion of better diet quality.

## 2. Materials and Methods

### 2.1. Chemicals and Reagents

Methanol (MeOH), acetonitrile (ACN), acetic acid (HAc), and sodium chloride (NaCl) were purchased from Merck (Darmstadt, Germany) and o-phthaldialdehyde (OPA) and trifluoroacetic acid (TFA) from Sigma-Aldrich (St. Louis, MO, USA). Type I water was obtained using a water purifier (Barnstead International, Dubuque, IA, USA). MeOH and ACN were of HPLC grade, and other reagents were of analytical grade. Phosphate-buffered saline solution was prepared adding 1.42 g of sodium phosphate dibasic, 0.245 g of potassium phosphate monobasic, 8.01 g of sodium chloride, 0.177 g of potassium chloride, and 90.15 mL of water and finally adjusted to pH 7.4. Chromatography columns were purchased from Agilent Technologies (Waldbronn, Germany).

Dried standards of AFB_1_, AFB_2_, AFG_1_, AFG_2_, OTA, DON, and FB_1_ were purchased from Sigma-Aldrich (St. Louis, MO, USA). FB_1_ standard was reconstituted in a mixture of ACN/H_2_O 1:1 *v*/*v*, while other standards were reconstituted in ACN. Aliquots were dried under a nitrogen stream (Domnick Hunter, Cleveland, OH, USA). Further reconstitution and dilutions were performed using a mixture of H_2_O/MeOH (1:1; *v*/*v*) for DON and ACN for the other standards. All standard solutions were stored at 4 °C.

### 2.2. Sampling Frame

Samples of whole-wheat flour (*Triticum aestivum* L.), brown rice (*Oriza sativa* L.), lupin (*Lupinus mutabilis* Sweet), quinoa (*Chenopodium quinoa* Willd.), and peanuts (*Arachis Hypogaea* L.) were collected following a stratified random sampling protocol, with 60% of samples drawn from local markets and 40% from supermarkets [38]. Samples were collected in the three most populated Ecuadorian cities (Quito, Guayaquil, and Cuenca). Cuenca and Quito are located in the Andean region at high altitudes (over 2.500 m.a.s.l.) and are characterized by subtropical highland climate with uniform rainfall. Guayaquil is a coastal city situated near the Pacific Ocean, with a tropical savanna climate [39]. Samples were collected during February to May 2018, which corresponded to the harvest in the dry season (April–September 2017) [40].

About 1 kg of each food, sold as bulk, was collected. For peanuts and whole-wheat flour, 45 samples (15 per city) were gathered; for brown rice, 47 samples (15 for Cuenca, 16 for Quito, and 16 for Guayaquil); for quinoa, 46 samples (15 for Cuenca, 16 for Quito, and 15 for Guayaquil); and for lupin, 36 samples (15 for Cuenca, 15 for Quito, and 6 for Guayaquil due to limited accessibility). All samples were collected and analyzed as dry raw material, except for the lupin samples from Cuenca, where lupin was only available in cooked form. Dry samples were packed in labeled, sealed paper bags, while wet samples (cooked lupin) were packed in airtight plastic bags. Samples (except for cooked lupin) were stored under dry conditions at 18 °C until analysis. Cooked lupin samples were immediately processed. Before analysis, each sample was milled to a fine, uniform particle size (20 Mesh), and it was thoroughly mixed, to ensure homogeneity and reduce the impact of uneven mycotoxin distribution.

### 2.3. Sample Treatment

The mycotoxins analyzed in each food matrix were selected based on occurrence and/or lack of data. OTA, FB_1_, and DON were studied in whole-wheat flour; OTA and AFs in peanuts; and OTA, FB_1_, and AFs in brown rice, quinoa, and lupin. Different sample extraction and clean-up procedures were carried out according to the food matrices and mycotoxin properties.

For FB_1_ extraction, a volume of 50 mL of a solvent mixture MeOH/H2O (75:25; *v*/*v*) was added to 20 g of milled sample. The suspension was shaken vigorously for 2 min; then, it was placed in a horizontal shaker (VWR, Allentown, PA, USA) at 300 rpm for 10 min. The mixture was filtered using a Whatman N° 4 filter. If necessary, pH was adjusted within 5.8–6.5 using 1M NaOH. The filtrate was centrifuged for 10 min at 2403 g (Hettich EBA 20, Tuttlingen, Germany). A volume of 10 mL of supernatant was applied to strong anion-exchange (SAX) clean-up cartridges (HyperSep, 3 mL/500 mg) (Thermo Scientific, Waltham, MA, USA), which were pre-conditioned with 5 mL of MeOH followed by 5 mL of MeOH/H_2_O (75:25; *v*/*v*) solution. The extract was passed at 1 mL min^−1^. The cartridge was washed with 8 mL of MeOH/H_2_O (75:25; *v*/*v*) followed by 3 mL of MeOH at 5 mL min^−1^. Then, the cartridge was dried by applying vacuum for 10 s. Finally, FB_1_ was eluted from the cartridge with 10 mL of MeOH/HAc (99:1; *v*/*v*) passing through by gravity. The eluate was evaporated at 30 °C using a dried bath (Thermo Scientific, Bremen, Germany) until the volume was reduced to about 1 mL; then, it was dried under a nitrogen stream. For derivatization, dried extract was reconstituted with 200 µL of MeOH. A volume of 200 µL of OPA reagent (40 mg in 1 mL of MeOH) was added, and it was vortexed for 30 s. The derivatized mixture was filtered (0.45 µm PVDF syringe membrane filters) and injected into the HPLC system within 1 min after adding the OPA reagent (mixture stable for up to 4 min).

For OTA extraction, a volume of 100 mL of the solvent mixture ACN/H2O (60:40; *v*/*v*) was added to 25 g of milled sample. The suspension was mixed in a horizontal shaker at 300 rpm for 30 min. The mixture was centrifuged for 10 min at 2403 g. A volume of 2 mL of extract was diluted with 22 mL of PBS solution. After vigorous shaking, the solution was applied to immunoaffinity clean-up (IAC) cartridges (Ochraprep, R-Biopharm Rhône, Glasgow, UK), which were previously brought to room temperature and pre-conditioned with 3 mL of PBS solution. The diluted extract was passed at 2–3 mL min^−1^ through the IAC cartridge. Then, it was washed with 10 mL of PBS solution followed by 10 mL of water at 5–6 mL min^−1^. The cartridge was dried by applying vacuum for 10 s. OTA was eluted from the cartridge with 1.5 mL of the mixture MeOH/acetic acid (98:2; *v*/*v*), passing through by gravity and by applying backflushing two times. An additional volume of 1.5 mL of H_2_O was applied into the cartridge. The final elute (3 mL) was filtered prior to HPLC injection.

For DON extraction, a volume of 100 mL of type I water was added to 25 g of milled sample and the suspension was mixed in a horizontal shaker at 300 rpm for 30 min. The mixture was centrifuged for 10 min at 1574 g. The IAC cartridges (Donprep, R-Biopharm Rhône, Glasgow, UK) were brought to room temperature. A volume of 2 mL of extract was passed through the cartridge at 1 mL min^−1^. Then, it was washed with 5 mL of H_2_O at 5–6 mL min^−1^. The cartridge was dried by applying vacuum for 10 s. DON was eluted from the cartridge with 0.75 mL of MeOH passing through by gravity and then by applying backflushing three times and adding a final volume of 0.75 mL of MeOH. The eluate was dried under a nitrogen stream. This was reconstituted using 1 mL of H_2_O/MeOH (90:10; *v*/*v*), vortexed for 30 s, and finally, filtered prior to HPLC analysis.

For AF extraction, a volume of 40 mL of the solvent mixture MeOH/H_2_O 80:20 (*v*/*v*) and 2 g of NaCl were added to 20 g of milled sample. The suspension was shaken vigorously by hand for 1 min and then in a horizontal shaker at 300 rpm for 30 min. The mixture was filtered using Whatman N° 4 filter paper, then centrifuged for 10 min at 1574 g. A volume of 5 mL of extract was diluted with 25 mL of PBS solution. The diluted extract was applied to immunoaffinity clean-up cartridges (Aflaprep, R-Biopharm Rhône, Glasgow, UK), which were previously brought to room temperature and conditioned with 1 mL of PBS solution. The extract was passed at 5 mL min^−1^. The cartridge was washed with 10 mL of PBS solution followed by 10 mL of water at a flow rate of 5–6 mL min^−1^. The cartridge was dried by applying vacuum for 10 s. AFs were eluted from the cartridge with 1 mL of MeOH passing through by gravity and then backflushed 3 times, adding a final volume of 0.5 mL of H_2_O. The eluate was dried under a nitrogen stream. For derivatization, dried extracts were reconstituted with 200 µL of N-hexane. A volume of 50 µL of TFA was added, and the mixture was vortexed for 30 s. The derivatized extract was dried under a nitrogen stream and was finally reconstituted with 1 mL of ACN/H_2_O (1:1; *v*/*v*). It was filtered prior to HPLC analysis.

### 2.4. HPLC Conditions and Analysis

Mycotoxins were analyzed on an Agilent 1200 HPLC system (Agilent Technologies, Santa Clara, CA, USA) consisting of a quaternary pump, a vacuum degasser, a thermostat autosampler, a column oven, a fluorescence detector, and a Diode Array Detector. A Zorbax Eclipse Plus C18 column (150 × 4.6 mm, 5 µm) (Agilent Technologies, Santa Clara, CA, USA) was used for FB_1_ and DON analyses, and a Zorbax Eclipse Plus C18 column (250 × 4.6 mm, 5 µm) was used for AF and OTA analyses.

For FB_1_, an isocratic elution using MeOH/0.1 M NaH_2_PO_4_ (75:25; *v*/*v*) at a flow rate of 1 mL min^−1^ was applied. The column oven was set to 25 °C. Fluorescence detection was carried out at Exc. 335 nm/Em. 440 nm. The injection volume was 20 µL.

For OTA, an isocratic elution was carried out using ACN/H_2_O/HAc (50:49:1; *v*/*v*/*v*) at a flow rate of 1 mL min^−1^. The column oven temperature was 25 °C. Fluorescence detection was performed at Exc. 247 nm/Em. 480 nm. The injection volume was 100 µL [34,41].

For DON, a gradient elution was applied using H_2_O/MeOH 90:10 (*v*/*v*) and MeOH as mobile phases A and B, respectively. The gradient started at 13% of B from 0 to 12 min; then, it increased to 49% of B at 14 min, and these conditions were maintained until 21 min. The column re-equilibrated until 27 min. The flow rate was 0.7 mL min^−1^, and the column was kept at 40 °C. DON was detected by a DAD (Diode Array Detector) at 220 nm. The injection volume was 10 µL.

AF analysis was carried out by applying a gradient elution using H_2_O/MeOH/HAc 89:10:1 (*v*/*v*/*v*), MeOH, and ACN as mobile phases A, B, and C, respectively. The concentration of C was kept constant during the run (16% of C). The gradient started with 25% of B for 1 min. Then, B increased from 25 to 70% at the 10th min, and it was kept for 3 min. The column was re-equilibrated to initial conditions at the 18th min. The flow rate was 1.2 mL min^−1^, and the column temperature was 40 °C. Fluorescence detection was set to Exc. 365 nm/Em. 455 nm for AFB_2_ and AFG_2_, and Exc. 365 nm/Em. 432 nm for AFB_1_ and AFG_1_. The injection volume was 5 µL.

Mycotoxins were identified by comparing the retention times with reference standards. Analytical methods were optimized considering the following parameters. Quantification and linearity were performed using calibration curves constructed in different concentration ranges for each mycotoxin, to cover a concentration level that was within the maximum permitted limits in the samples according to European Regulation 2023/915/EC [42]. For AFs, a 0.5–50 µg L^−1^ range was used; for DON, 50–1000 µg L^−1^; for FB_1_, 10–100 µg L^−1^; and for OTA, 0.1–100 µg L^−1^. The limits of detection (LODs) and limits of quantification (LOQs) were determined using the formula LOD = 3 × S_bl_/S, where S_bl_ is the standard deviation of the intercept and S is the slope of the respective linear regression calibration curve. The LOQs were calculated using LOQ = 6 × S_bl_/S. To assess linearity, calibration curves of standard solutions in pure solvents at different concentration levels were constructed [43]. Recovery experiments for each mycotoxin were performed in triplicate by spiking a test sample before extraction at 20 µg L^−1^.

## 3. Results and Discussion

### 3.1. HPLC Method Performance

The calibration curves of all analyzed mycotoxins showed good linearity (R > 0.99). Retention times were 7.0 min for FB_1_, 8.35 min for DON, 10.4 min for OTA, 5.6 min for AFB_1_, 4.65 min for AFB_2_, 3.76 min for AFG_1_, and 3.16 min for AFG_2_, considering a general retention time window of 0.05 min. Average recoveries were 99% for FB_1_, 88.4% for DON, 88% for OTA, 81% for AFB_1_, 87% for AFB_2_, 62% for AFG_1_, and 76% for AFG_2_. LODs and LOQs are presented in Table 1.

Although LC-MS/MS is the most selective and sensitive methodology for mycotoxin analysis, HPLC methods were employed in this study due to instrumentation availability. Even though this is a limitation, the obtained detection limits were low enough to accurately assess the contamination level in all foods below the MPLs (Table 2).

### 3.2. Maximum Permitted Levels in Minimally Processed Foods

There are no national or regional regulations on mycotoxin MPLs in the analyzed foods. Thus, the MPLs of European Regulation 2023/915/EC [42] were adopted as reference for comparison (Table 2). This regulation includes the MPLs for peanuts and wheat intended for human consumption, whereas no limits for quinoa, lupin, and brown rice are available. Therefore, MPLs for comparable food categories were selected based on processing type and compositional similarities. All foods evaluated in this study undergo some degree of sorting or minor processing before consumption. Brown rice and quinoa are typically washed and cooked; lupin is commonly washed, debittered, boiled, and salted; whole-wheat flour is baked into bread or pastries; and peanuts are usually roasted, salted, or sugar-coated. Those processing steps support the selection of MPLs corresponding to products intended for processing. Thus, quinoa was compared to unprocessed cereals, reflecting its similar post-harvest processing. Lupin was aligned with seeds as a proxy category.

For brown rice, specific MPLs are set only for AFs. Then, FB_1_ was compared with the MPLs set for maize for final consumer. However, the regulation defines MPLs only for the sum of FB_1_ + FB_2_; a more conservative approach was adopted by using 50% of the total MPL (i.e., 500 µg kg^−1^), which is a stricter threshold than the typical 70–80% contribution of FB_1_ [44,45,46], ensuring a margin of safety in the absence of disaggregated regulatory values. The MPL for OTA is only available for peanuts; then, the MPL for cereals in general was adopted to compare the contamination levels of the other foods.

### 3.3. Mycotoxin Co-Occurrence in Minimally Processed Traditional Foods in Ecuador

The natural occurrence of FB_1_ and DON associated with field contamination and of OTA and AFs produced during storage conditions was investigated. Contamination rates, overall and per city, were categorized in three groups: (i) ancestral foods (quinoa and lupin) (Table 3); (ii) peanuts (Table 4); and (iii) whole grains (brown rice and wheat flour) (Table 5).

**Figure 1:**
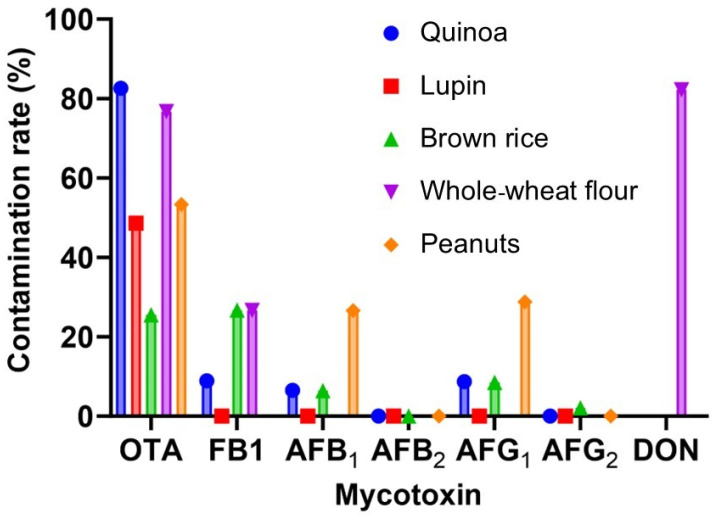
Overall mycotoxin (FB_1_, DON, OTA, and AFs) contamination rates in quinoa, lupin, peanuts, brown rice, and whole-wheat flour.

Overall contamination reached up to 59.8% of the analyzed samples (38.4% with one mycotoxin and 21.5% with two or more mycotoxins) (Figure 1). OTA was the most prevalent mycotoxin (in 82.6% of quinoa, 76.7% of whole-wheat flour, 53.3% of peanuts, 48.6% of lupin, and 25.5% of brown rice). DON was found in above 80% of the whole-wheat flour samples. FB_1_ was detected in above 25% of the brown rice and whole-wheat flour samples and in 9% of the quinoa samples. AFB_1_ and AFG_1_ showed similar prevalence (around 6.5 and 8.5%, respectively) in quinoa and brown rice and about 27% prevalence in peanuts. AFB_2_ tested negative in all analyzed samples, while AFG_2_ was scarcely present in peanuts (2.2%).

**Figure 2:**
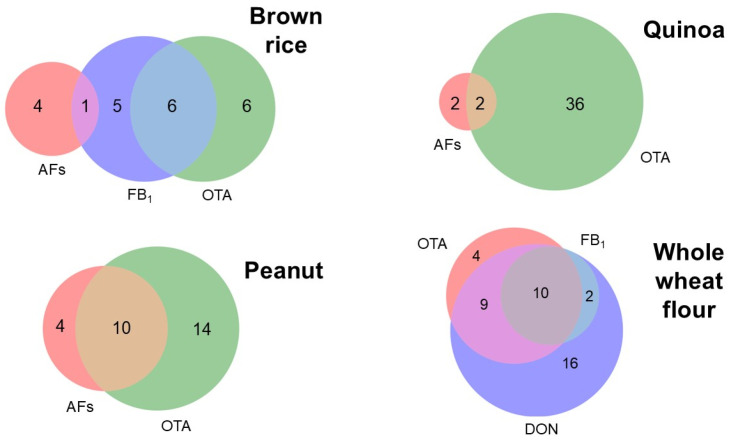
Venn diagrams of mycotoxin contamination and co-occurrence combinations in brown rice, quinoa, peanuts, and whole-wheat flour. Shown numbers in each color represent the number of contaminated samples per mycotoxin, while numbers within mixed colors represent co-contamination.

Overall co-occurrence with two or more mycotoxins was 21.5% (47 samples) (Supplementary Materials). Mycotoxin co-occurrence was mostly prevalent in whole-wheat flour (44.7%), followed by peanuts (27.7%), brown rice (19.1%), and quinoa (8.5%). No co-occurrence in lupin samples was observed. In general, the most common mycotoxin combination among co-contaminated samples was OTA and AFs (26%), followed by OTA, DON, and FB_1_ (21%); OTA and DON (19%); AFB_1_ and AFG_1_ (15%); OTA and FB_1_ (13%); FB_1_ and DON (4%) and AFB_1_, AFG_1_ and FB_1_ (2%) (Figure 2). Of these combinations, only OTA and AFs suggest a post-harvest contamination pattern, especially in peanuts (10 out of 13 co-contaminated samples). Other combinations address the importance of enhancing fungal control in the pre- and post-harvest stages. This natural co-occurrence of mycotoxins suggests the presence of environmental conditions that favor the simultaneous or successive contamination of food products with different fungal species, as well as moisture uptake by stored grains from damp soils or walls, wind-driven rain, lack of adequate ventilation, or the mixing of dry and wet grains [47,48].

### 3.4. Mycotoxin Occurrence in Quinoa and Lupin

In Ecuador, quinoa is produced at a medium scale in the north-central highland provinces. It is collected and stored in silos, from where it is usually distributed in smaller bulks across the country [49]. In this study (Table 2), limited contamination of FB_1_ was found in quinoa (8.9%), without surpassing the MPL; meanwhile OTA was highly prevalent across cities (69 to 100%), and 21.1% of samples were above the MPL, with similar ranges among sampling sites. Out of the AFs analyzed, AFB_1_ and AFG_1_ were detected, yet the sum of them did not surpass the MPL. The contamination pattern observed in quinoa suggested post-harvest contamination during storage in silos or insufficient drying, which should reach less than 14% to avoid a favorable environment for fungal growth, especially for toxigenic species such as Aspergillus and Penicillium [48,50].

Mycotoxin contamination is poorly documented for quinoa. AFB_1_, FB_1_, and OTA were investigated in Peruvian quinoa samples by targeted metabolomics. Contrary to our study, only FB_1_ traces were detected in 7 out of 21 samples, all below the LOQs and MPLs [24]. Another study investigated *Fum1*, a gene involved in the production of type B fumonisins (including FB_1_). *Fum1* was found in 1 out of 11 samples of Peruvian quinoa. Also, a small number of samples were tested for DON and zearalenone (ZEN). DON was tested negative (two samples), while ZEN was found in one sample of quinoa (out of one) [51]. In addition, the emerging mycotoxin beauvericin (BEA) has been reported in 16 out of 27 samples of Andean quinoa [24]. Those patterns might suggest that quinoa can also be contaminated in the pre-harvest stage.

In Ecuador, lupin is produced at a small scale in the north highland provinces. For consumption, lupin beans require initial drying to reduce the water content to 12–14% [52], followed by a treatment to reduce the inherent toxic alkaloid content (called debittering), which involves soaking, cooking, and washing for several hours using large volumes of salted water. Debittered beans are finally dried again and stored [53,54]. After this second drying process, lupin becomes more susceptible to fungal growth and further contamination with post-harvest mycotoxins. Partially, it could be caused by the reduced antifungal activity of alkaloids after debittering [53,54]. In this study, FB_1_ and AFs were not detected in lupin samples. OTA was detected in almost half of the samples (all cooked lupin, and 2 out of 21 dry lupin samples), but the above-MPL contamination rate was very low (5.7%). Different contamination patterns were observed across sampling sites. No contamination was observed in Guayaquil samples, whereas in Quito only 2 out of 15 samples were contaminated and displayed the highest OTA concentrations, 2 and 4 times the MPL of 5 µg kg^−1^. It must be considered that OTA is produced at high water activity (≥0.93) [55], suggesting that the humidity in the Andean region might be enough for post-harvest mycotoxin production. In Cuenca, since no dried samples were available, cooked lupin beans were analyzed, with all samples (out of 15) testing positive but under the MPL. This lower contamination level might be result of the additional processing. OTA is highly heat-stable, requiring temperatures higher than those achieved during boiling to obtain significant reductions [56]. However, the partial elimination of OTA can be expected during the debittering of the alkaloids before cooking in water, since it becomes soluble in aqueous alkaline solutions [57]. Nonetheless, this potential reduction mechanism requires further evaluation. On the other hand, differences across cities could be related to diverse food distribution fluxes from production provinces, where non-standardized first-drying processes take place.

The contamination of lupin with some Fusarium mycotoxins has been previously described. A 2005 study analyzing German foods revealed the presence of nivalenol (one out of nine), HT-2 and T-2 toxin (one out of nine), and 15-monoacetoxyscirpenol (two out of nine) [58]. In addition, the occasional presence of phomopsins has been documented [59,60]. To the best of our knowledge, no study has been conducted investigating lupin contamination with OTA, FB_1_, and AFs in the Andean and Latin America region.

### 3.5. Mycotoxin Occurrence in Peanut Samples

Peanuts are produced in the coastal region. They are usually consumed peeled, roasted, and/or pressed. This processing patterns might result in lower mycotoxin occurrence, but seed inner layers make this food matrix susceptible to fungal development, particularly during storage [61].

The results of the analyzed mycotoxins in peanuts samples are presented in Table 3. In this food, only the post-harvest mycotoxins, OTA and AFs, were analyzed.

The overall prevalence of OTA reaches more than half of the samples, but only 2.2% surpasses the MPL. Prevalence was higher in the samples from the studied Andean regions (Cuenca and Quito), which aligns with the suggested weather conditions for OTA production, i.e., mild temperatures (15–25 °C) [62,63]. Worldwide, OTA contamination in peanuts is very variable. For instance, OTA was found in 32% of Argentinian peanut samples within 0.5 to 170 µg kg^−1^ [64], while in Côte d’Ivoire samples, it was reported as low as in a range from 0.203 to 0.642 µg kg^−1^ [65].

On the other hand, AFB_1_ and AFG_1_ were the main aflatoxins detected in the peanut samples, without exceeding the MPLs. However, one sample showed an AFG_1_ concentration of 44.7 µg kg^−1^, which represents 7.7% of the total aflatoxin-contaminated samples (1 out of 13) exceeding the MPL for the sum of aflatoxins (AFt). These AF rates are in line with previous studies. However, considerable differences in concentration ranges associated with geographical and seasonal fluctuations have been reported [66,67]. In the Latin American context, several studies had been conducted in Brazilian peanuts and peanut sub-products. AF concentrations ranged from 7 to 116 µg kg^−1^, with lower contamination in the most processed foods [68,69]. Processing seems to reduce not only AFs but also OTA contamination in various peanut products [70,71]. The co-occurrence of OTA and AFs was observed in the studied peanut samples, similarly to other reports [72]. This co-occurrence supports the need for precise interventions, implementing efficient mycotoxin post-harvest monitoring.

### 3.6. Mycotoxin Occurrence in Whole Grains: Brown Rice and Whole-Wheat Flour

In Ecuador, rice is produced at a large scale across the coastal region. After harvesting, it is dried until lower humidity (<14%) is reached prior to milling. Then, it is stored in silos and later distributed to nearby provinces. These conditions make rice a commodity very susceptible to fungal contamination in the pre- and post-harvest stages.

In this study, FB_1_, OTA, and AFs were found in brown rice, but none of the mycotoxin contamination exceeded the MPLs (Table 4). The highest contamination rates were observed in Cuenca, with 8 out of 15 samples testing positive for FB_1_ and 11 out of 15 samples testing positive for OTA. These findings suggest that pre- and post-harvest conditions may be in favor of mycotoxin production. Overall, only the Quito samples showed rather low contamination with AFs (below 9%). In Ecuador, mycotoxin co-occurrence in rice has been previously reported by our group [34], showing that 49% of paddy rice and 7% of polished rice samples were contaminated with at least one mycotoxin, with AFB_1_ and FB_1_ exceeding the MPLs in paddy rice. Moreover, it was suggested that dehusking can decrease the mycotoxin load from paddy rice to polished rice. This may also have an impact on brown rice, as it is a mid-dehusking product [73]. In this line, a higher level of OTA (0.2 μg kg^−1^) in brown rice in comparison to white and parboiled rice (0.15 µg kg^−1^) has been previously reported [74]. Fungal stress resulting from reduced moisture content plays a pivotal role in stimulating mycotoxin production [75]. Differences in contamination rates between regions suggest possible impacts from environmental and storage conditions at silos, especially over prolonged periods [76]. Similarly to our findings, AFs were detected in brown rice (*n* = 9) in all samples ranging from 0.03 to 8.33 µg kg^−1^ in the Philippines [77], where rice production and consumption is considerably high. Another study on brown rice from Guyana showed AF contamination with a mean of 23.95 µg kg^−1^ [74]. In contrast, a study on Swedish brown rice found that the samples (*n* = 6) tested negative for OTA and AFs [78].

Ecuador is the one of main producing (9.1% of the Gross Domestic Product share) and consuming countries of rice in the Andean region. According to the Ecuadorian Ministry of Agriculture, Livestock, Aquaculture, and Fisheries (MAGAP), the average consumption per capita is approximately 53.2 kg per year [79]; thus, a more comprehensive monitoring of potential hazards associated with rice contamination is advisable. At this time, no study has been conducted investigating Ecuadorian brown rice mycotoxin contamination.

Ecuadorian wheat production does not meet the national demand, and subsequently, it is mostly imported. However, there is a limited supply of whole-wheat flour that is locally produced mainly in the north-central highlands [80]. In this study, notable contamination rates of DON (82.2%) and OTA (76.7%) and lower FB_1_ rates (26.7%) were found (Table 3). While none of the samples surpassed the MPL for OTA, 50% of samples exceeded the MPL for FB_1_, predominantly in Cuenca, where values reached more than 1400 µg kg^−1^. DON is known to be the most abundant mycotoxin in wheat worldwide [81]. In our study, most of the samples were contaminated with DON, with contamination 37.8% above the MPL. Noteworthily, the Cuenca samples displayed the highest contamination level, with concentrations nearly four times above the MPL (almost 4500 µg kg^−1^), and Quito samples had concentrations reaching above 2500 µg kg^−1^. These rates are similar to previous findings reported in China, where DON in whole-wheat flour reached concentrations above 900 µg kg^−1^ [82]. In contrast, while 13 out of 15 Guayaquil samples tested positive for DON, none of them were above the MPL. DON contamination in Ecuadorian wheat products has been previously reported by our group at lower concentrations [34], indicating that processing could reduce the mycotoxin load from whole-wheat to refined flour and further processed products. The observed contamination pattern suggests the need for fungal control in the pre-harvest stage, especially for the intended consumption of whole grains that are minimally processed [83].

The samples of minimally processed foods evaluated in this study were collected from retail places from three regions in Ecuador. Considering that the final storage at retail stores in small bulks might be short, no regional climatic differences in mycotoxin contamination were assessed in this study. Instead, the importance of the food production, storage, drying, and distribution stages on mycotoxin production was emphasized. However, the influence of storage conditions in different regions, as well as that of handling practices and insect infestation, on mycotoxin contamination in foodstuffs at the final selling points should be further assessed, especially in humid and warm regions [84].

Whole grains are actively promoted as part of a healthy and sustainable diet since they are low-fat staple foods rich in dietary fiber, micronutrients, and functional phytochemicals contained in the outer layer and germ fractions [85]. However, the known nutritional benefits of whole-grain consumption might not compensate the potential chemical contamination risks associated with the outer bran [86].

## 4. Conclusions

This study represents the first report on the natural occurrence of seven mycotoxins in a range of minimally processed traditional foods in Ecuador, with a particular focus on ancestral foods (quinoa and lupin), peanuts, and whole grains (brown rice and whole-wheat flour). Overall contamination with at least one mycotoxin was 59.8% (38.4% with one mycotoxin and 21.5% with two or more mycotoxins). The most frequent co-occurrence combinations were OTA and AFs, and OTA, DON, and FB_1_. OTA emerged as the most prevalent mycotoxin in all analyzed foods, particularly in quinoa, with a considerable number of samples exceeding maximum permissible levels. Whole-wheat flour also exhibited important contamination rates and levels of DON and FB_1_.

Different contamination patterns are highlighted. These were not strictly associated with the sampling region but might be linked with the food distribution fluxes and handling. These findings underscore the importance of enhancing fungal control in the pre- and post-harvest stages, particularly for whole grains and other minimally processed foods. Continuous monitoring and targeted interventions that include affordable and pragmatic strategies such as drying techniques and improved storage practices could contribute to mitigating mycotoxin contamination in these minimally processed nutritious foods. Although these foods are not highly consumed, these are usually promoted as rich sources of dietary fiber, and their consumption might increase over time. Therefore, it is highly recommended to increase studies on exposure assessment by combining population consumption data to evaluate actual risk in the Ecuadorian diet.

This study serves as a foundational step in understanding and addressing mycotoxin occurrence in these minimally processed Ecuadorian foods, contributing valuable insights to healthy diet promotion, further exposure assessment, and food sovereignty.

## Figures and Tables

**Table 1:** LODs and LOQs for the analyzed mycotoxins (all expressed in µg kg^−1^).

Mycotoxin	LOD	LOQ
FB_1_	3.15	6.30
OTA	0.07	0.14
DON	9.47	18.94
AFB_1_	0.06	0.13
AFB_2_	0.20	0.40
AFG_1_	0.10	0.20
AFG_2_	0.16	0.32

FB_1_, fumonisin B_1_; OTA, ochratoxin A; DON, deoxynivalenol; AFB_1_, aflatoxin B_1_; AFB_2_, aflatoxin B_2_; AFG_1_, aflatoxin G_1_; AFG_2_, aflatoxin G_2_; AFt, total aflatoxins (sum of AFB_1_ + AFB_2_ + AFG_1_ + AFG_2_).

**Table 2:** Reference maximum permitted levels (MPLs) allowed in foods for human consumption (all expressed in µg kg^−1^).

Mycotoxin	MPLs (2023/915/EC)
FB_1_	1000 (FB_1_ + FB_2_) ^a^
OTA	5 (peanuts); 3 (others) ^b^
DON	600 ^c^
AFB_1_	2 (quinoa) ^d^; 5 (rice ^e^); 8 (peanuts and lupins ^f^)
AFB_2_	-
AFG_1_	-
AFG_2_	-
AFt	4 (quinoa) ^d^; 10 (rice ^e^); 15 (peanuts and lupins ^f^)

FB_1_, fumonisin B_1_; OTA, ochratoxin A; DON, deoxynivalenol; AFB_1_, aflatoxin B_1_; AFB_2_, aflatoxin B_2_; AFG_1_, aflatoxin G_1_; AFG_2_, aflatoxin G_2_; AFt, total aflatoxins (sum of AFB_1_ + AFB_2_ + AFG_1_ + AFG_2_). ^a^ MPL set for maize, maize-based foods, and milling products for placing on the market for the final consumer. ^b^ MPL set for cereals for placing on the market for the final consumer. ^c^ MPL set for milling products of cereals. ^d^ MPL set for cereals and products derived from cereals. ^e^ MPL set for maize and rice to be subjected to sorting or other physical treatment before placing on the market for the final consumer or use as an ingredient in food. ^f^ MPL set for hazelnuts and Brazil nuts to be subjected to sorting or other physical treatment before placing on the market for the final consumer or use as an ingredient in food.

**Table 3:** Mycotoxin (FB_1_, OTA, and AFs) occurrence in ancestral foods samples: quinoa and lupin. Contamination rates (CRs), rates over maximum permitted levels (% > MPLs), means, standard deviations (SDs), and ranges, overall and per city. Means and ranges are in µg kg^−1^.

	Quinoa (*n* = 46)						Lupin (*n* = 36)					
	Overall		Per City				Overall		Per City			
Mycotoxin	CR (%)	% > MPL	City	Positive/Total	Mean ± SD	Range	CR (%)	% > MPL	City	Positive/Total	Mean ± SD	Range
FB_1_	8.9	0	C	1/15	21.3 ^a^	-	n.d.	-	C	0/15	-	-
			Q	3/16	8.2 ± 1.7	6.3–9.6	-	-	Q	0/15	-	-
			G	0/15	-	-	-	-	G	0/6	-	-
OTA	82.6	21.1	C	15/15	2.3 ± 3.0	0.78–10.7	48.6	5.7	C	15/15	1.1 ± 0.14	0.89–1.4
			Q	11/16	3.5 ± 2.6	0.98–16.7	-	-	Q	2/15	15.9 ± 9.0	9.6–22.3
			G	12/15	4.5 ± 4.8	1.3–9.3	-	-	G	0/6	-	-
AFB_1_	6.5	0	C	0/15	-	-	n.d.	-	C	0/15	-	-
			Q	2/16	0.03 ± 0.002	0.03–0.03	-	-	Q	0/15	-	-
			G	1/15	0.05 ^a^	-	-	-	G	0/6	-	-
AFB_2_	n.d.	-	C	0/15	-	-	n.d.	-	C	0/15	-	-
			Q	0/16	-	-	-	-	Q	0/15	-	-
			G	0/15	-	-	-	-	G	0/6	-	-
AFG_1_	8.7	-	C	0/15	-	-	n.d.	-	C	0/15	-	-
			Q	2/16	0.14 ± 0.05	0.11–0.18	-	-	Q	0/15	-	-
			G	2/15	0.07 ± 0.02	0.06–0.08	-	-	G	0/6	-	-
AFG_2_	n.d.	-	C	0/15	-	-	n.d.	-	C	0/15	-	-
			Q	0/16	-	-	-	-	Q	0/15	-	-
			G	0/15	-	-	-	-	G	0/6	-	-
AFt	-	0		-	-	-	-	-		-	-	-

FB_1_, fumonisin B_1_; OTA, ochratoxin A; AFB_1_, aflatoxin B_1_; AFB_2_, aflatoxin B_2_; AFG_1_, aflatoxin G_1_; AFG_2_, aflatoxin G_2_; AFt, total aflatoxins (sum of AFB_1_ + AFB_2_ + AFG_1_ + AFG_2_); C, Cuenca; Q, Quito; G, Guayaquil; n.d., non-detected. ^a^ Unique value.

**Table 4:** Mycotoxin (OTA and AFs) occurrence in peanut samples. Contamination rates (CRS), rates over maximum permitted levels (% > MPLs), means, standard deviations (SDs), and ranges, overall and per city. Means and ranges are in µg kg^−1^.

	Peanuts (*n* = 45)					
	Overall		Per City			
Mycotoxin	CR (%)	% > MPL	City	Positive/Total	Mean ± SD	Range
OTA	53.3	2.2	C	15/15	1.2 ± 0.23	0.9–1.7
			Q	7/15	4.2 ± 5.6	1.2–16.8
			G	2/15	2.9 ± 2.4	1.2–4.6
AFB_1_	26.7	0	C	3/15	0.23 ± 0.22	0.06–0.47
			Q	5/15	0.19 ± 0.28	0.05–0.69
			G	4/15	0.05 ± 0.02	0.03–0.07
AFB_2_	n.d.	-	C	0/15	-	-
			Q	0/15	-	-
			G	0/15	-	-
AFG_1_	28.9	- ^b^	C	3/15	0.77 ± 1	0.13–1.9
			Q	5/15	0.57 ± 0.98	0.06–2.3
			G	5/15	4.1 ± 13.5	0.07–44.7
AFG_2_	2.2	- ^b^	C	1/15	0.14 ^a^	
			Q	0/15	-	-
			G	0/15	-	-
AFt	- ^c^	7.7		-	-	-

OTA, ochratoxin A; AFB_1_, aflatoxin B_1_; AFB_2_, aflatoxin B_2_; AFG_1_, aflatoxin G_1_; AFG_2_, aflatoxin G_2_; AFt, total aflatoxins (sum of AFB_1_ + AFB_2_ + AFG_1_ + AFG_2_); C, Cuenca; Q, Quito; G, Guayaquil; non-detected. ^a^ Unique value. ^b^ MPLs are not established for AFG1 and AFG2 alone. The % > MPL for AFt is reported. ^c^ The %CR for AFt is not reported, as it is calculated as the sum of individual aflatoxins.

**Table 5:** Mycotoxin (FB_1_, OTA, DON, and AFs) occurrence in whole-grain samples: brown rice and wheat flour. Contamination rates (CRs), rates over maximum permitted levels (% > MPLs), means, standard deviations (SDs), and ranges, overall and per city. Means and ranges are in µg kg^−1^.

	Brown Rice (*n* = 47)						Whole-Wheat Flour (*n* = 45)					
	Overall		Per City				Overall		Per City			
Mycotoxin	CR (%)	% > MPL	City	Positive/Total	Mean ± SD	Range	CR (%)	% > MPL	City	Positive/Total	Mean ± SD	Range
FB_1_	25.5	0	C	8/15	106 ± 16.3	74.8–122.8	26.7	50.0	C	9/15	812.3 ± 407.5	384.5–1430.9
			Q	4/16	14.1 ± 2.9	10.8–13.0	-	-	Q	1/15	11.04 ^a^	-
			G	0/16	-	-	-	-	G	2/15	5.9 ± 1.3	4.4–6.1
OTA	25.5	0	C	11/15	0.86 ± 0.08	0.79–1.06	76.7	0	C	15/15	0.96 ± 0.19	0.77–1.6
			Q	0/16	-	-	-	-	Q	8/15	1.4 ± 0.39	0.9–2.1
			G	1/16	1.12 ^a^	-	-	-	G	n.a.		
DON	n.a.		-	-	-	-	82.2	37.8	C	14/15	1878 ± 1181	166.2–4445.0
			-	-	-	-	-	-	Q	10/15	881.7 ± 741.1	139.0–2057.3
			-	-	-	-	-	-	G	13/15	242 ± 48	115.0–286.8
AFB_1_	6.4	0	C	0/15	-	-	-	-	-	-	-	-
			Q	3/16	0.07 ± 0.05	0.03–0.11	-	-	-	-	-	-
			G	0/16	-	-	-	-	-	-	-	-
AFB_2_	n.d.	-^b^	C	0/15	-	-	-	-	-	-	-	-
			Q	0/16	-	-	-	-	-	-	-	-
			G	0/16	-	-	-	-	-	-	-	-
AFG_1_	8.5	- ^b^	C	0/15	-	-	-	-	-	-	-	-
			Q	3/16	0.89 ± 1.02	0.17–1.6	-	-	-	-	-	-
			G	1/16	0.07 ^a^		-	-	-	-	-	-
AFG_2_	2.1	- ^b^	C	0/15	-	-	-	-	-	-	-	-
			Q	0/16	-	-	-	-	-	-	-	-
			G	1/16	0.07 ^a^	-	-	-	-	-	-	-
AFt	- ^c^	0		-	-	-	-	-	-	-	-	-

FB_1_, fumonisin B_1_; OTA, ochratoxin A; DON, deoxynivalenol; AFB_1_, aflatoxin B_1_; AFB_2_, aflatoxin B_2_; AFG_1_, aflatoxin G_1_; AFG_2_, aflatoxin G_2_; C, Cuenca; Q, Quito; G, Guayaquil; n.d., non-detected; n.a., non-assessed. ^a^ Unique value. ^b^ MPLs are not established for AFG1 and AFG2 alone. The % > MPL for AFt is reported. ^c^ The %CR for AFt is not reported, as it is calculated as the sum of individual aflatoxins.

## Data Availability

The original contributions presented in the study are included in the article, and further inquiries can be directed to the corresponding author.

## Supplementary Materials

The following supporting information can be downloaded at: https://www.mdpi.com/article/10.3390/foods14152621/s1, Table S1: Dataset of mycotoxin contamination in quinoa, lupin, peanuts, brown rice and whole-wheat flour.

## Abbreviations

The following abbreviations are used in this manuscript:

AFB_1_	Aflatoxin B_1_
AFB_2_	Aflatoxin B_2_
AFG_1_	Aflatoxin G_1_
AFG_2_	Aflatoxin G_2_
AFs	Aflatoxins (AFB_1_, AFB_2_, AFG_1_, AFG_2_)
AFt	Total aflatoxins (AFB_1_ + AFB_2_ + AFG_1_ + AFG_2_)
OTA	Ochratoxin A
FB_1_	Fumonisin B_1_
DON	Deoxynivalenol
MPL	Maximum permitted level
RP-HPLC	Reversed-Phase High-Performance Liquid Chromatography
DAD	Diode Array Detector
LOD	Limit of Detection
LOQ	Limit of Quantification
